# ITV versus mid-ventilation for treatment planning in lung SBRT: a comparison of target coverage and PTV adequacy by using in-treatment 4D cone beam CT

**DOI:** 10.1186/s13014-020-01496-5

**Published:** 2020-03-03

**Authors:** J. Bellec, F. Arab-Ceschia, J. Castelli, C. Lafond, E. Chajon

**Affiliations:** 1 Medical Physics Department, Centre Eugène Marquis, avenue de La Bataille Flandres Dunkerque – CS 44229, F-35042 Rennes, France; 2 Radiotherapy Department, Centre Eugène Marquis, avenue de La Bataille Flandres Dunkerque – CS 44229, F-35042 Rennes, France; 3 Inserm, U1099, F-35000 Rennes, France; 4 Université de Rennes-1, LTSI, F-35000 Rennes, France

**Keywords:** Lung cancer, ITV, Mid-ventilation, 4D-CBCT, Respiratory motions, Stereotactic body radiation therapy (SBRT), Geometric uncertainties, Planning strategy

## Abstract

**Background:**

The internal target volume (ITV) approach and the mid-ventilation (MidV) concept are the two main respiratory motion-management strategies under free breathing. The purpose of this work was to compare the actual in-treatment target coverage during volumetric modulated arctherapy (VMAT) delivered through both ITV-based and MidV-based planning target volume (PTV) and to provide knowledge in choosing the optimal PTV for stereotactic body radiotherapy (SBRT) for lung lesions.

**Methods and materials:**

Thirty-two lung cancer patients treated by a VMAT technique were included in the study. For each fraction, the mean time-weighted position of the target was localized by using a 4-dimensional cone-beam CT (4D-CBCT)-based image guidance procedure. The respiratory-correlated location of the gross tumor volume (GTV) during treatment delivery was determined for each fraction by using in-treatment 4D-CBCT images acquired concurrently with VMAT delivery (4D-CBCT_in-treat_). The GTV was delineated from each of the ten respiratory phase-sorted 4D-CBCT_in-treat_ datasets for each fraction. We defined target coverage as the average percentage of the GTV included within the PTV during the patient’s breathing cycle averaged over the treatment course. Target coverage and PTVs were reported for a MidV-based PTV (PTV_*MidV*_) using dose-probabilistic margins and three ITV-based PTVs using isotropic margins of 5 mm (PTV_*ITV + 5mm*_), 4 mm (PTV_*ITV + 4mm*_) and 3 mm (PTV_*ITV + 3mm*_). The in-treatment baseline displacements and target motion amplitudes were reported to evaluate the impact of both parameters on target coverage.

**Results:**

Overall, 100 4D-CBCT_in-treat_ images were analyzed. The mean target coverage was 98.6, 99.6, 98.9 and 97.2% for PTV_*MidV*_, PTV_*ITV + 5mm*_, PTV_*ITV + 4mm*_ and PTV_*ITV + 3mm*_, respectively. All the PTV margins led to a target coverage per treatment higher than 95% in at least 90% of the evaluated cases. Compared to PTV_*ITV + 5mm*_, PTV_*MidV*_, PTV_*ITV + 4mm*_ and PTV_*ITV + 3mm*_ had mean PTV reductions of 16, 19 and 33%, respectively.

**Conclusion:**

When implementing VMAT with 4D-CBCT-based image guidance, an ITV-based approach with a tighter margin than the commonly used 5 mm margin remains an alternative to the MidV-based approach for reducing healthy tissue exposure in lung SBRT. Compared to PTV_*MidV*_, PTV_*ITV + 3mm*_ significantly reduced the PTV while still maintaining an adequate in-treatment target coverage.

## Background

Stereotactic body radiotherapy (SBRT) is considered a standard treatment for inoperable early-stage non-small lung cell cancer (NSLCC) and oligometastases [1]. In SBRT, a highly accurate tumor location is critical, and advanced image guidance and delivery techniques are required to strictly confine the dose deposition to the tumor. One treatment solution for lung SBRT is volumetric modulated arc therapy (VMAT) combined with respiration-correlated 4D cone-beam computed tomography (4D-CBCT) image guidance. VMAT is recognized to reduce the delivery time and risk of intrafraction deviations in terms of both setup errors and organ motion [2]. At present, respiration-correlated 4D-CBCT is considered one of the optimal volumetric image-guided technologies for lung SBRT treatment [3]. With 4D-CBCT, the mean position, trajectory, and shape of a moving tumor can be verified in the treatment unit and thus, the respiration-induced geometrical uncertainties for mobile targets can be reduced [4].

When implementing VMAT with a 4D-CBCT image guidance protocol for lung SBRT, the two main treatment planning strategies used are the internal target volume (ITV) approach and the mid-ventilation (MidV) concept [5]. With the ITV-based strategy, the planning target volume (PTV) is designed to encompass the whole respiratory tumor motion area using an additional generic margin to account for other treatment uncertainties (i.e., setup, mechanical +/− delineation uncertainties). With the MidV-based strategy, the PTV is designed around the mean time-weighted tumor position using a probabilistic margin calculation that considers respiratory motion as a random positioning error [6].

Because of the ablative intent of SBRT, minimizing the PTV margins is essential for ensuring that a high dose will be delivered to the target while limiting the exposure to the surrounding healthy tissue [7]. Target coverage and margin adequacy should be ideally evaluated at the time of beam delivery to account for actual localization errors and potential breathing pattern modifications during treatment. With the recent availability of in-treatment respiration-correlated 4D-CBCT (4D-CBCT_in-treat_) in clinics, 4D-CBCT projections can be acquired concurrently with VMAT delivery, and thus the actual 4D target locations during treatment can be visualized at the end of each fraction [8, 9]. 4D-CBCT_in-treat_ imaging is a reliable tool for validating the PTV margins [10]. However, thus far, no study has analyzed these in-treatment 4D images to compare margin adequacy among different treatment planning strategies for lung SBRT.

The aim of the current study was to compare PTVs and target coverage derived from in-treatment 4D-CBCT images for both MidV-based and ITV-based strategies for VMAT lung SBRT. Target coverage was assessed geometrically by reporting the actual percentage of tumor volume included in the PTV during beam delivery throughout the treatment. The data derived from this work provide knowledge in choosing the optimal PTV margins for VMAT lung SBRT when a 4D-CBCT image guidance procedure is implemented.

## Methods and materials

### Patients

Thirty-two patients with lung cancer or metastasis were included in this study. The lesions were located in the lower lobe (20 cases), upper lobe (10 cases) and median lobe (2 cases). All patients underwent frameless 4D image-guided lung SBRT in our institution between 2016 and 2018.

### Treatment planning and delivery

All patients were treated with a VMAT technique on a Versa HD™ linear accelerator (Elekta, Crawley, UK). Image guidance was performed using the kilovoltage CBCT X-Ray Volume Imaging (XVI) system (Elekta, Crawley, UK) equipped with the Symmetry™ 4D-CBCT and Intrafraction modules (Elekta, Crawley, UK). A 6D HexaPOD™ evo RT treatment couch (Elekta, Crawley, UK) was used for patient repositioning. All patients were scanned and treated in a head-first supine position on a comfortable mattress using an arm and knee support without a dedicated stereotactic body frame or abdominal compression. For each patient, a planning 4D-CT was acquired using a Brilliance Big Bore CT scanner (Philips, Eindhoven, the Netherlands). During CT acquisition, a Pneumo Chest Bellows system (Philips, Eindhoven, the Netherlands) was employed to record the respiratory signal under free breathing. Ten respiratory phases CT data with a 2 mm thickness were then retrospectively reconstructed using phase sorting. Treatment planning was performed using the Pinnacle^3^ v9.6 treatment planning system (Philips Radiation Oncology Systems, Milpitas, USA). The gross tumor volume (GTV) was delineated on ten respiratory phase-sorted 4D-CT datasets using the lung window. No margins for microscopic extension were added (i.e. CTV = GTV). All patients were treated using an ITV-based strategy with an additional ITV-to-PTV margin of 4 mm. The ITV was generated by performing the union of the 10 phase-sorted GTVs. The VMAT treatment plans were designed using a single arc of 200°, while avoiding the contralateral lung with either a flattened 6 MV photon beam or a 6 MV flattening filter free (FFF) photon beam. A total dose of 54 Gy in 3 fractions was prescribed for peripheral targets, and total doses of 48 to 54 Gy in 4 to 6 fractions were prescribed for centrally located targets and targets in contact with the chest wall. The dose was prescribed to an isodose surface that encompassed at least 95% of the PTV. The maximum dose within the PTV was approximately 125% of the prescribed dose.

At treatment delivery, after aligning the patient on the treatment couch, a first respiration correlated 4D-CBCT was acquired. No external respiratory system was required for phase sorting: the breathing signal relied on the motion of the diaphragm in cranio-caudal axis directly extracted from the 2D cone beam projection data acquired during the gantry rotation [4]. The 2D projections and the respiratory signal were sorted in 10 phase bins to reconstruct ten respiratory phases 4D-CBCT datasets.

After bony anatomy registration, the mean time-weighted target position was identified by performing an automatic local rigid registration of the 10 phase-sorted 4D-CBCT datasets to the MidV-CT dataset on a region of interest surrounding the ITV and excluding bony structures. The MidV-CT referred to the respiratory phase-sorted CT derived from the planning 4D-CT and showed the GTV closest to its mean time-weighted position. A post-correction 3D-CBCT scan was then acquired after couch corrections to ensure that the target was within the PTV prior to VMAT delivery. The 4D-CBCT_in-treat_ was then concurrently acquired with VMAT delivery. At the end of the fraction, 4D target motions during beam delivery were visualized on the 4D-CBCT_in-treat_ to assess that the target remained within the PTV during VMAT delivery.

### PTV margins

For each patient, four PTV margins were evaluated, including a MidV-based PTV setting (PTV_*MidV*_) and three ITV-based PTV margins (PTV_*ITV*_).

The PTV_MidV_ was designed around the GTV closest to the time-weighted position (GTV_MidV_) delineated on the MidV-CT images [11]. The PTV margins were individually calculated for each patient in the left-right (LR), anteroposterior (AP) and craniocaudal (CC) directions using the margin equation from van Herk et al. [12, 13] (Eq.1).

1 $$ {M}_{PT{V}_{MidV}}=2.5\sqrt{\left({\Sigma}_{baseline}^2+{\Sigma}_{MidV}2+{\Sigma}_{iso}2\right)}+0.84\sqrt{\left({\sigma}_{baseline}^2+{\sigma}_{TM}^2+{\sigma}_p^2\right)}-0.84{\sigma}_p $$

The margin value ${M}_{PT{V}_{MidV}}$ calculated with Eq. 1 ensures that the GTV of 90% of patients receives the prescribed dose, considering a prescription isodose level of 80% [13]. Σ and σ denote the standard deviations of the systematic localization errors and random localization errors, respectively. The baseline localization errors (Σ_baseline_ and σ_baseline_) were derived from the patient group with the intrafraction baseline displacements observed on the 4D-CBCT_in-treat_ images (i.e., distance between the mean time-weighted tumor position during beam delivery and the mean time-weighted tumor position during planning). The residual mean time-weighted tumor localization error during planning (Σ_MidV_) was derived from the 4D-CT of the patient group. The random contribution due to breathing motion (σ_TM_) was individually determined for each patient from the planning 4D-CT using one-third of the peak-to-peak tumor motion amplitude [12]. σ_p_ denotes the standard deviation of the dose gradient (penumbra) in the lung tissue, which was considered to be 6.4 mm [13]. The residual imaging-to-treatment isocenter misalignment of the treatment unit was also included (Σ_iso_). Target delineation uncertainties were not included in the PTV margins. Table 1 summarizes the values of the systematic and random errors used to calculate the dose-probability-based margins for the MidV-based strategy. For our patient cohort, without any respiratory tumor motion, the MidV-based PTV margin necessary to cover the intrafraction baseline displacements and residual mechanical errors was LR 5.0 mm, AP 4.9 mm and CC 7.5 mm.

**Table 1:** Systematic (Σ) and random (σ) target localization errors of the patient group and related dose-probability-based PTV margins calculated for the MidV approach. Baseline error refers to the residual localization error of the mean time-weighted target position. Iso error refers to the error related to the nonperfect coincidence between imaging and treatment isocenters. MidV error refers to the residual error of localizing the mean time-weighted target position in the MidV CT dataset

	LR (mm)	AP (mm)	CC (mm)
Σ_baseline_	1.9	1.9	2.8
σ_baseline_	1.5	1.5	2.3
Σ_iso_	0.4	0.3	0.3
Σ_MidV_	0.1	0.3	0.5
Margins [range]	[5.0–5.2]	[5.0–6.0]	[7.5–12.2]

For the ITV-based PTV margins, the ITV was defined as the union of the 10 phase-sorted GTVs delineated on the 4D-CT phase-sorted datasets. Three PTVs were set using generic isotropic margins of 5 mm (PTV_*ITV + 5mm*_), 4 mm (*PTV*_*ITV + 4mm*_) and 3 mm (*PTV*_*ITV + 3mm*_).

### Target coverage evaluation

**Fig. 1:**
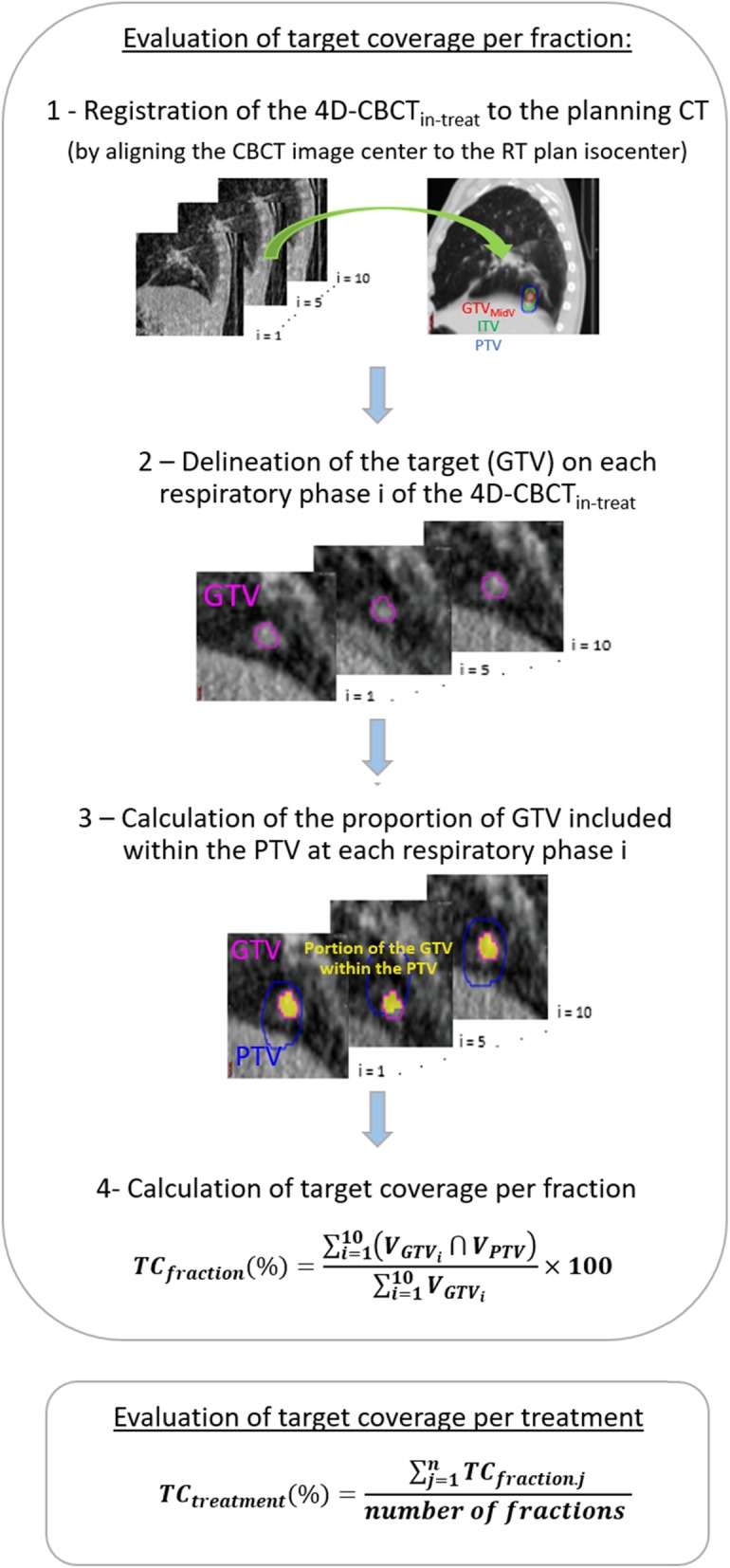
Workflow of the calculations for in-treatment target coverage (TC)

For each patient, the ten respiratory phase-sorted 4D-CBCT_in-treat_ images reconstructed for each fraction, RT plan in DICOM format and PTV were exported to MIM software version 6.5 (MIM Software Inc., Cleveland OH, USA). The GTV was delineated from ten respiratory phase-sorted 4D-CBCT_in-treat_ datasets for each fraction by a single senior radiation oncologist. We defined target coverage as the average proportion of the GTV included within the PTV over the respiratory phases. Target coverage was calculated for each of the four PTV settings using the workflow described in Fig. 1. For each evaluated case, the target coverage per fraction was first evaluated. The effective target coverage per treatment was then deduced in each case by averaging the target coverage over all fractions. We defined a target coverage per treatment of 95% as the threshold for adequate target coverage [14, 15].

### Data analysis

PTVs and in-treatment target coverage were compared for the different PTV margins and according to the respiratory motion amplitude vector defined on the planning 4D-CTs of all patients. In our analysis, “motion amplitude vector” referred to the vector length calculated from the tumor peak-to-peak amplitudes measured in the LR, AP and CC directions [11].

Additionally, intrafraction baseline displacement during beam delivery and the difference in the motion amplitude vectors between treatment and planning were reported for each fraction to identify a potential correlation between target coverage and both of these parameters. Baseline displacement described the displacement of the mean time-weighed position of the target relative to the expected mean time-weighed position of the target defined during planning. Baseline displacement was defined as the distance (3D vector) between the centroid of the mean time-weighted tumor calculated from the 4D-CBCT_in-treat_ and the centroid of the mean time-weighted tumor defined during planning. The difference in the motion amplitude vectors was defined as the difference between the motion amplitude vector of the 4D-CBCT_in-treat_ and that of the planning 4D-CT. This parameter was chosen to quantify breathing pattern modifications during beam delivery.

Statistical analysis was performed using SPSS v20.0 software. Comparisons were performed with paired Wilcoxon signed-ranked tests. Differences were considered statistically significant at *p < 0.05*.

## Results

The median GTV was 2.1 cc (range: 0.3 cc–13.1 cc). The median respiratory amplitude vector on the planning 4D-CT was 8.6 mm (range: 1.3 mm–26.4 mm).

Overall, 100 4D-CBCT_in-treat_ images (i.e., 1000 phase-sorted CBCT datasets) were delineated and analyzed.

### PTVs

**Fig. 2:**
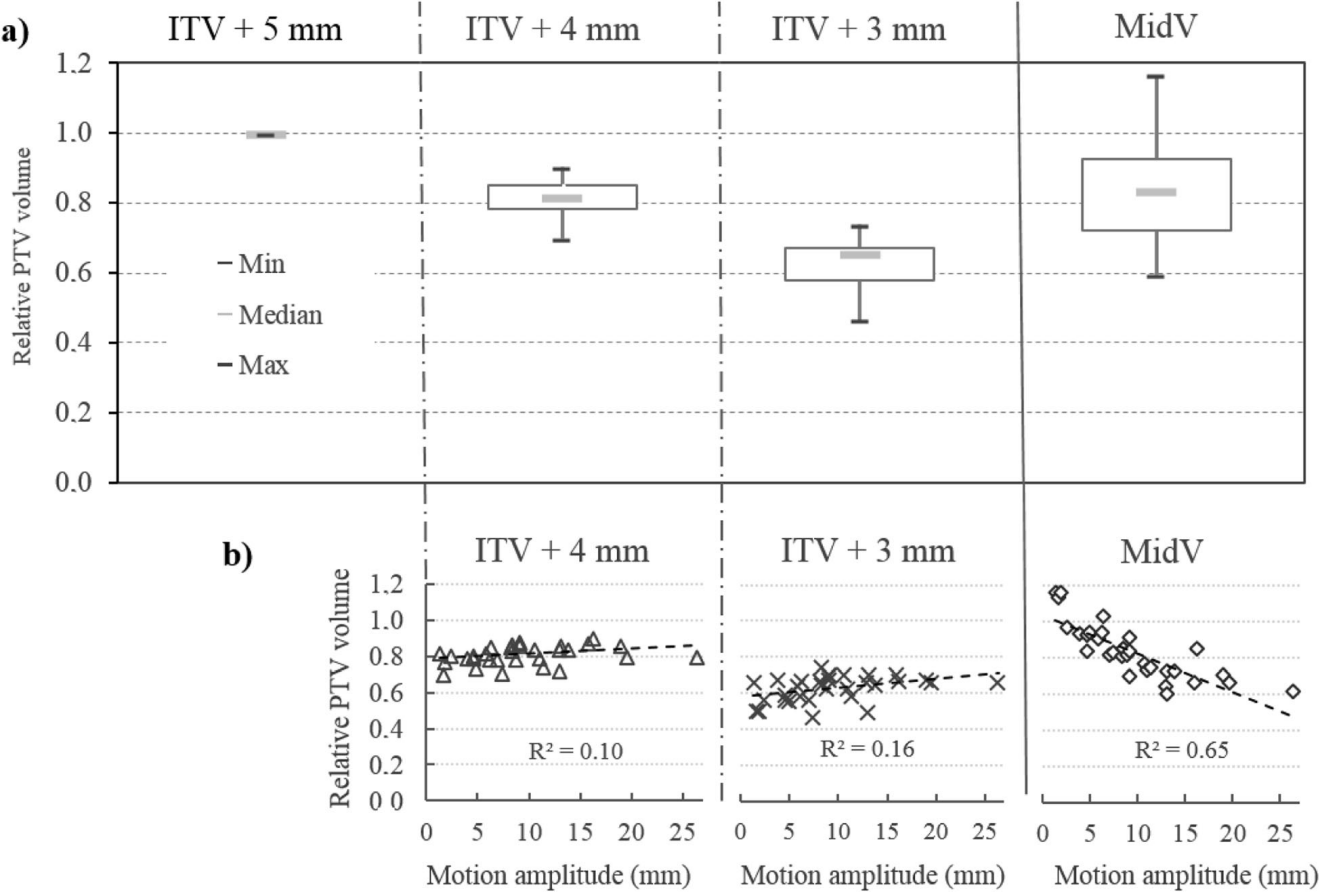
**a** Relative PTVs normalized to PTV_ITV + 5mm_ for the different PTV margins. Relative PTVs are plotted in (**b**) as a function of target motion amplitude vector. PTV, as a function of respiratory amplitude, clearly shows a strong correlation with the MidV-based strategy

The relative PTVs of the different PTV margins are reported in Fig. 2a. The PTV volumes of each patient were normalized to PTV_*ITV + 5mm*_ to overcome interpatient PTV variations. When compared to PTV_*ITV + 5mm*_, PTV_MidV_ led to a significant mean PTV reduction of 16% (*p* < 0.001). The volume reduction from PTV_*ITV + 4 mm*_ was not significantly different from that observed with PTV_*MidV*_ (*p* = 0.36). PTV_*ITV + 3mm*_ showed the smallest PTV with a significant mean reduction of 37% relative to PTV_*ITV + 5mm*_ (*p* < 0.001) and was significantly smaller than PTV_*MidV*_ with a mean reduction of 25% (*p* < 0.001).

The relative PTV as a function of respiratory amplitude is shown in Fig. 2b. The correlation evaluated by Pearson’s product-moment coefficient shows a strong correlation between respiratory amplitude and relative PTV_MidV_ (R^2^ = 0.65). In contrast, a very weak correlation was observed for respiratory motion amplitude and ITV-based PTV margins (R^2^ < 0.2).

### In-treatment target coverage

**Fig. 3:**
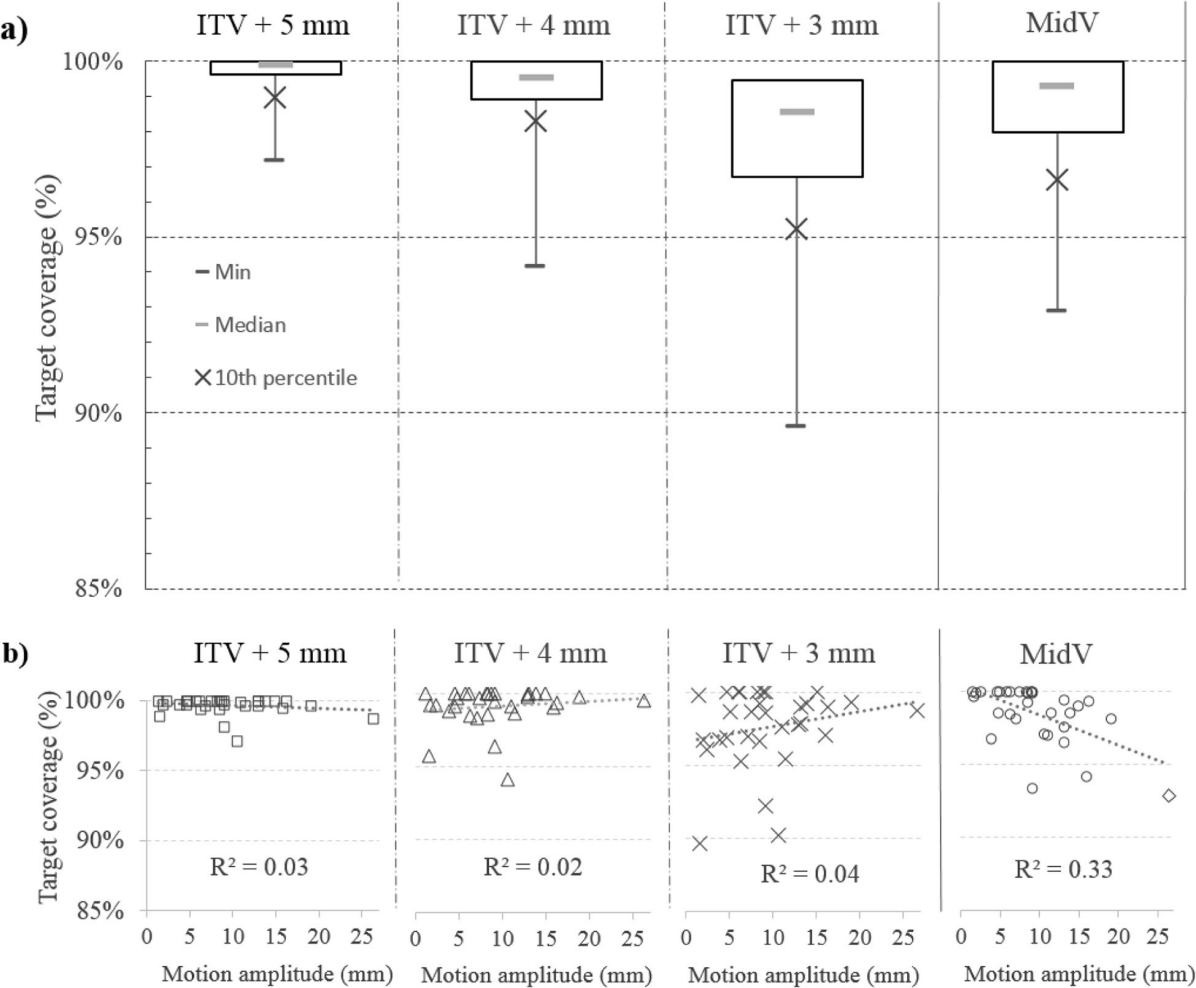
**a** Target coverage per treatment for the different PTV margins. Target coverage per treatment is plotted in (**b**) as a function of the tumor motion amplitude vector. On boxplots (**a**), the cross represents the 10th percentile and highlights the minimum target coverage obtained for at least 90% of the patients

Figure 3a shows target coverage per treatment for the different PTV margins. The mean target coverage per treatment was 99.6, 98.9, 97.2 and 98.6% for_,_ PTV_*ITV + 5mm*_, PTV_*ITV + 4mm*_, PTV_*ITV + 3mm*_*and* PTV_*MidV,*_ respectively; the minimum target coverage was 96.7, 93.5, 89.7 and 93.3% for PTV_*ITV + 5mm*_ PTV_*ITV + 4mm,*_ PTV_*ITV + 3mm*_*and* PTV_*MidV,*_ respectively. All the PTV settings led to a target coverage per treatment higher than 95% for at least 90% of the patients. The target coverage was not significantly different between PTV_*ITV + 4 mm*_ and PTV_*MidV*_ (*p* = 0.40).

Target coverage as a function of respiratory amplitude is reported in Fig. 3b. No correlation was found between the motion amplitude vector and ITV-based PTV (R^2^ < 0.05). A weak correlation was observed between the motion amplitude vector and the MidV-based PTV (R^2^ = 0.33): target coverage tended to decrease as the motion amplitude increased.

### Impact of intrafraction baseline displacements and motion amplitude variability on target coverage

**Fig. 4:**
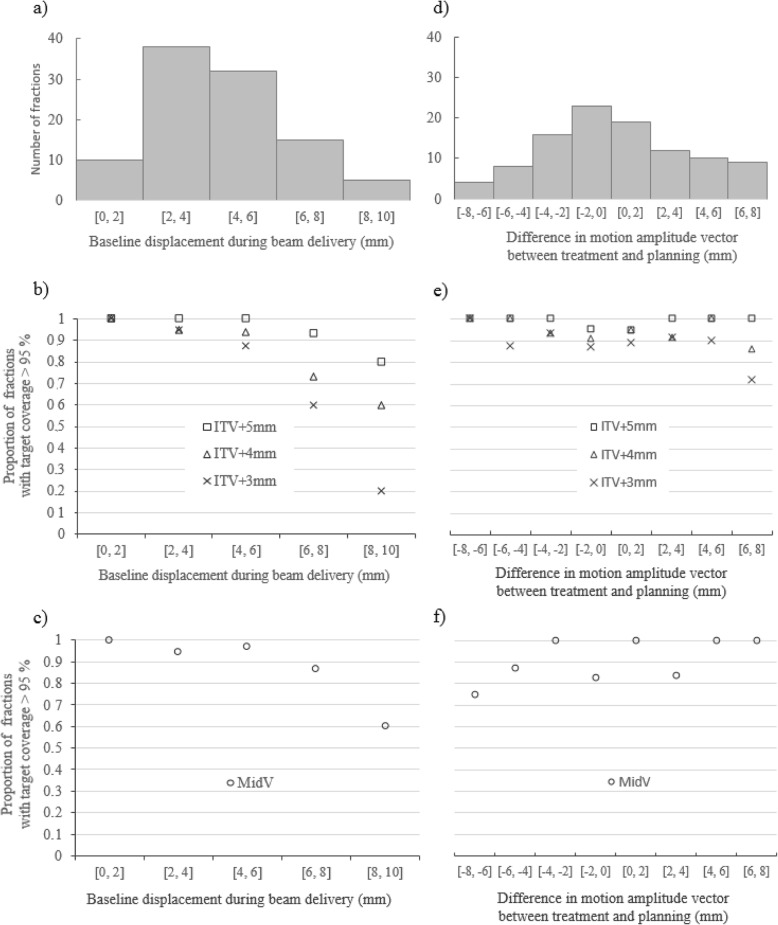
**a** Histogram of the intrafraction baseline displacements observed during beam delivery (vector length) indicating that in 80% of fractions, the baseline displacements were smaller than 6 mm. An analysis of the impact of these baseline displacements on the target coverage is reported in (**b**) and (**c**) for the ITV-based PTV and the MidV-based PTV, respectively. **d** Histogram of the differences in motion amplitude vectors between treatment and planning shows that in 81% of fractions, the motion amplitude vector is consistent with that of planning at +/−4 mm. The impact of these motion amplitude differences on target coverage is reported in (**e**) and (**f**) for the ITV-based PTV and the MidV-based PTV, respectively

A histogram of baseline displacements that occurred during beam delivery is reported in Fig. 4a., which shows that in 80% of all fractions, the baseline displacements were less than 6 mm (vector length). Figure 4b and c show the relative number of fractions with a target coverage > 95% as a function of baseline displacement for ITV-based PTVs and the MidV-based PTV, respectively. The loss of target coverage was strongly impacted by baseline displacements larger than 6 mm (vector). The slope values of the linear regression lines in Fig. 4b. are − 0.05, − 0.10 and − 0.19 for PTV_*ITV + 5mm*_, PTV_*ITV + 4mm*_ and PTV_*ITV + 3mm*_, respectively, indicating that the impact of baseline displacements increased when the PTV margins decreased.

A histogram of the differences in motion amplitude vectors between treatment and planning is shown in Fig. 4d and indicates that compared to that observed during the planning 4D-CT, the target motion amplitude during beam delivery was higher for 49% of the fractions and was smaller in 51% of the fractions. The differences ranged from − 8 to + 8 mm. In total, 70% of the motion amplitude differences were between +/− 4 mm. Figure 4e and f show the relative number of fractions with target coverage > 95% as a function of the difference in motion amplitude vectors for ITV-based PTVs and the MidV-based PTV, respectively. As the variations in the motion amplitude vectors did not exceed 6 mm relative to those during planning (in 91% of all fractions), no correlation was clearly identified, regardless of the PTV used. Loss of target coverage was noticeable only for PTV_*ITV + 4 mm*_ and PTV_*ITV + 3 mm*_ with the largest variations in the motion amplitude vector (i.e., ranging from 6 to 8 mm), yet the impact was moderate: 85 and 70% of these fractions still had a target coverage higher than 95% for PTV_*ITV + 4mm*_ and PTV_*ITV + 3mm*_, respectively.

## Discussion

In the present work, geometric target coverage and margin adequacy were evaluated for two common planning strategies usually implemented with a respiration-correlated 4D-CBCT-image-guided lung SBRT.

The most innovative aspect of our study was that target coverage was evaluated by using in-treatment 4D-CBCT images acquired concurrently with beam delivery. The actual respiration-correlated 4D positions of the target during beam delivery were therefore considered. To the best of our knowledge, all previous studies comparing respiratory motion management strategies in lung SBRT were performed exclusively with pretreatment data [6, 16, 17]. In our study, the evaluation incorporated all residual localization errors and account for the effective breathing patterns of the patient during treatment. Thus, the present work could be considered a “end-to-end” comparison of PTV margins.

In SBRT, high doses are delivered in a few fractions to maximize tumor control. Thus, SBRT may increase toxicity in the adjacent healthy tissues. Therefore, minimizing the irradiated volume without compromising target coverage is crucial. The optimal PTV could be defined as the volume that maintains the smallest margins as prudently possible without sacrificing target coverage [7]. As expected, the ITV-based PTV boundaries using a 5 mm margin resulted in the largest PTV but resulted in excellent in-treatment target coverage for all evaluated cases. Previous studies reported that a MidV approach could significantly reduce PTVs and therefore reduce lung tissue exposure compared to an ITV-based PTV using a 5 mm margin [6, 11, 12, 16, 17]. In the present work, the PTV_*MidV*_ was on average 16% smaller than PTV_*ITV + 5mm*_, which is similar to the study results of Wanet et al. [16]. With the MidV approach, the margins were delineated to ensure that the GTV of 90% of patients received the prescribed dose [12, 13]; in our patient cohort, an adequate target coverage (> 95%) was achieved in 29/32 evaluated cases (i.e., 90.6% of cases), which was consistent with the MidV margin calculations. Based on these observations, the MidV approach can be considered an optimal planning strategy for mobile lung lesions when no active motion management technique is available [6, 11]. Nevertheless, to the best of our knowledge, previous published studies comparing MidV and ITV strategies for lung SBRT were all performed using ITV-based margins equal to or larger than 5 mm [6, 11, 17]. Our study demonstrated that an ITV-based approach remained an appealing alternative to the MidV approach when considering tighter margins than the commonly used 5 mm value. Indeed, by reducing the margin from 5 to 4 mm, the resulting differences between the ITV-based PTV and the MidV-based PTV (*p* = 0.36) and target coverage (*p* = 0.40) were not statistically significant. By reducing the margin to 3 mm, the ITV-based PTV was on average smaller (− 25%; *p* < 0.001) than the MidV-based PTV while still enabling adequate target coverage (> 95%) for more than 90% of the patients.

With the MidV approach, the PTV margins are weakly influenced by respiratory amplitude, unlike in the ITV approach [6, 11]. When compared to an ITV-based PTV, the relative MidV PTV has a tendency to decrease as the tumor motion amplitude increases. This characteristic of the MidV approach is illustrated in Fig. 2b: when compared to the PTV_*ITV + 4mm*_, the relative MidV-based PTV was smaller by approximately 10% for a 5 mm motion amplitude and was smaller by approximately 35% for a 20 mm respiratory amplitude. In contrast, by using an ITV-based approach with tighter PTV margins than 5 mm, the PTV was not correlated with tumor motion amplitude and thus was similar for all patients regardless of tumor motion amplitude.

With a MidV-based PTV, in-treatment target coverage tended to decrease when the motion amplitude increased (Fig. 3b), which can be explained by the inherent nature of the approach, since respiratory motions are not fully included in the PTV margins and thus, the tumor may not be fully located within the PTV during a small portion of the breathing cycle [6]. In the case of small tumors with a large motion amplitude, Wanet et al. [16] used a Monte Carlo simulation to report that a MidV-based PTV might result in underdosage. In the present report, the same observation was made: the lowest target coverage with a MidV-based PTV was observed for the patient with a small peripheral lesion and the largest tumor motion amplitude (i.e., 26 mm). With an ITV-based strategy, on the other hand, in-treatment target coverage was not correlated with respiratory motions because respiratory motions are fully included in the PTV.

The recent ESTRO-ACROP guidelines indicate that the ITV-based strategy is still the most widely accepted approach for treatment planning in lung SBRT [5]. From a practical point of view, implementing an ITV approach does not require a population-based margin equation, and generic margins can be applied. In contrast, before implementing a MidV-based strategy, margin formalism requires an assessment of residual geometric uncertainties based on institution-specific protocols by analyzing an in-treatment dataset. Thus, when starting a lung SBRT program, the ITV concept should be prioritized, at least for the first several treatments. Moreover, Ehrbar et al. [17] reported that the current 3D dose calculation method used in treatment planning systems remains an accurate estimation of the effective dose delivered to mobile tumors with an ITV-based strategy, whereas the effective dose delivered tended to be overestimated when using the MidV strategy. Based on these observations, an ITV approach using small margins remains an appealing alternative to the MidV approach in lung SBRT when no active motion management technique is used.

The analysis of respiratory motion amplitude on the 4D-CBCT_in-treat_ image confirmed that using a single 4D-CT scan for planning did not capture the actual tumor motions observed at the time of treatment [18]. These deviations might be partially explained by physiological factors, such as patient nervousness. However, this finding could also be explained by technological factors: a fan-beam 4D-CT captures only a snapshot of the patient’s breathing pattern, whereas a 4D-CBCT records the patient’s breathing pattern averaged over a 3 min window. However, the motion amplitude variability had a very limited impact on target coverage and seems to be mostly compensated for by the PTV margins regardless of the PTV approach used. For instance, even when considering a difference in motion amplitude between 6 to 8 mm (less than 10% of the fractions analyzed) and the smallest PTV (i.e. PTV_*ITV + 3mm*_) an adequate target coverage (> 95%) was still achieved for the majority of these fractions. Intrafraction baseline displacements, on the other hand, are a well-known and significant component of PTV margins for ensuring adequate target coverage in lung SBRT [19, 20]. These findings are clearly illustrated in Fig. 4b by changing the ITV-based PTV margins from 5 to 3 mm. Moreover, an MidV-based PTV seems to be slightly less affected by the baseline displacements than an ITV-based PTV of similar size (i.e., PTV_*ITV + 4 mm*_), which can be explained by the fact that systematic and random errors related to baseline displacements were explicitly incorporated in the margin formalism of the MidV approach.

A limitation of the present study was that target coverage was based on geometric criteria not on dosimetric criteria. A 4D accumulated dose analysis should ideally be performed to quantify the actual dose coverage during beam delivery taking into account the tissue density effect and the potential interplay between the target motion and the temporal aspect of VMAT delivery. However, dose calculation and accumulation based on CBCT data is still an unresolved issue that is beyond the scope of the present work.

## Conclusion

When using a single-arc VMAT technique with 4D-CBCT image guidance, the ITV-based approach using tighter PTV margins than the commonly used 5 mm value remains an alternative to the MidV approach for limiting exposure to the surrounding healthy tissues in lung SBRT. Compared to the MidV PTV, an ITV-based PTV with a 3 mm margin significantly reduced the PTV in all patients while still maintaining adequate in-treatment target coverage for at least 90% of patients.

## Data Availability

All data analysed during this study are included in this published article.

## Abbreviations

4D-CBCT: 4-Dimensionnal Cone beam Computed Tomography
4D-CBCT_in-treat_: In-treatment 4-Dimensionnal Cone beam Computed Tomography
4D-CT: 4-Dimensionnal Computed Tomography
CTV: Clinical target volume
GTV: Gross target volume
ITV: Internal target volume
MidV: Mid-ventilation
PTV: Planning target volume
PTV_ITV + x mm_: ITV-based planning target volume with x mm margin
PTV_MidV_: Mid-ventilation-based planning target volume
